# Development of a shoulder muscle feedback controller for human body models

**DOI:** 10.3389/fbioe.2026.1694396

**Published:** 2026-03-05

**Authors:** Emma Larsson, Jason Fice, Johan Iraeus, Jonas Östh, Bengt Pipkorn, Johan Davidsson

**Affiliations:** 1 Department of Mechanics and Maritime Sciences, Chalmers University of Technology, Gothenburg, Sweden; 2 Volvo Car Corporation, Gothenburg, Sweden; 3 Autoliv Research, Vårgårda, Sweden

**Keywords:** active human body model, driver, muscle controller, pre-crash, shoulder muscles

## Abstract

**Introduction:**

State-of-the-art finite element human body models (FE HBMs) with active muscle controllers can predict occupant kinematics during braking and steering, which are typical pre-crash interventions aiming at avoiding crashes. Information about the pre-crash occupant kinematics can be used in the design of systems that influence the occupant position in the pre-crash phase and the interaction between the occupant and the restraints in both the pre- and in-crash phases. For driver HBMs, active shoulder muscles are required to reproduce the load between the steering wheel and the torso. The shoulder is the most freely moving joint in the body, and the stability of the shoulder complex depends on muscle activity. Thus, intermuscular load sharing cannot be determined solely from the geometrical location of the muscle because other muscles co-contract to maintain stability during the movement. The aims of this study were to implement a new controller, which introduces load sharing based on physical tests with volunteers, into the shoulder of an FE-HBM and to compare its performance with that of volunteers subjected to dynamic elbow loading.

**Methods:**

A new shoulder muscle controller for use in FE-HBMs was developed, including directionally dependent intermuscular load sharing based on recorded muscle activity from volunteers. The controller performance was evaluated by simulating a volunteer experiment, exposing the subjects to dynamic loading of their elbow in eight directions.

**Results:**

Elbow kinematics were compared between the model and volunteers. A sensitivity study was also performed to evaluate the controller gains. The model successfully predicted peak elbow displacements for all loading directions.

**Discussion:**

One limitation in the current study was the use of a submodel and a simplified experimental setup. In a braking or steering maneuver, head and torso inertia would introduce forces to the shoulder, instead of forces introduced in the elbow as in this study. Because these two scenarios are mechanically similar, a simplified approach was used instead, as this allowed for an experiment where the force magnitude and direction could be easily controlled. Hence, the developed shoulder muscle controller is ready to be implemented and evaluated in a full-body active FE-HBM exposed to driver maneuvers.

## Introduction

Automotive crashes are often preceded by an evasive maneuver such as braking, steering or combinations of these (Hault-Dubrulle et al., 2011a; Scanlon et al., 2015; Riexinger and Gabler, 2018; Riexinger et al., 2019), which can influence the occupant posture and position (Ólafsdóttir et al., 2013; Östh et al., 2013; Huber et al., 2015; Fice et al., 2021; Hedenstierna et al., 2008; Ghaffari et al., 2018; Reed et al., 2019), and in turn, potentially influence the injury outcome in a subsequent crash (Bose et al., 2010; Hault-Dubrulle et al., 2011b; Donlon et al., 2020). For drivers, who typically keep their hands on the steering wheel, forces transferred through the arms may play an important role in the occupant position during the evasive maneuver.

The forces from the steering wheel are transferred to the torso via the arms and the shoulder joints. The shoulder joint is a combination of two joints, the glenohumeral joint, which allows for large humeral rotations, and the scapulothoracic joint, which allows for both translations and rotations of the scapula, together forming the most freely moving joint in the body (Marieb and Hoehn, 2019). The stability of the shoulder joint and its capacity to transfer loads from the hands to the torso are maintained by muscle contraction (Marieb and Hoehn, 2019).

Finite element human body models (FE-HBMs) are valuable tools in the design of safer vehicles, increasingly being used in the automotive industry (Yang and Chou, 2015). To study occupant kinematics during braking and steering, FE-HBMs have been equipped with active muscle controllers (Östh et al., 2015). While these models can predict passenger kinematics in braking and steering (Kato et al., 2017; Kato et al., 2018; Devane et al., 2019; Larsson et al., 2019), only a few models predict driver kinematics in these maneuvers (Kato et al., 2017; Devane et al., 2019). One difference between a driver and a passenger is the ability to actively engage the arms, and thus, active shoulder control is required to model a driver holding on to the steering wheel in evasive maneuvers.

A few driver models incorporating active shoulders are available (Östh et al., 2015; Kato et al., 2017; Kato et al., 2018; Devane et al., 2019). The SAFER HBM uses a single proportional–integral–derivative (PID) controller to activate muscles to reduce sagittal plane angle changes of the humerus (Östh et al., 2015). The muscles are grouped as flexors or extensors, and all muscles in each group receive the same level of activation. The Total Human Model for Safety (THUMS) v5 driver model (Kato et al., 2017) utilizes three PID controllers for the projected angular displacements of the humerus in a local coordinate system and three PID controllers for the hand forces. In addition, two PID controllers are used to control the scapula displacements (Kato et al., 2018). In the THUMS v5 model, the intermuscular load sharing was pre-defined using published anatomical data (Kato et al., 2017). The musculature from THUMS v5 was carried over to THUMS v6 and is included in the latest THUMS v7 that has been developed for reclined occupant analysis (Matsuda et al., 2023). The Global Human Body Models Consortium (GHBMC) M50-OS v2 + Active Driver Model uses a similar controller configuration as the THUMS model, but with intermuscular load sharing based on the model’s anatomical function rather than anatomical data (Devane et al., 2019).

Although some driver models use shoulder muscle controllers to respond to the displacements of the humerus, none are based on human shoulder muscle physiological data derived from volunteer experiments. As the stability of the shoulder complex depends on muscle activity, the intermuscular load sharing cannot be determined solely from the geometrical location of the muscle because other muscles co-contract to maintain stability during the movement (Nikooyan et al., 2012; Kian et al., 2019). One study of an active shoulder model found that using recorded muscle activity data in the model (Nikooyan et al., 2012) improved predicted glenohumeral joint reaction forces by 45% compared with *in vivo* joint force measurements. This suggests that human muscle activity measurements can be used to achieve more realistic intermuscular load sharing in the shoulder complex.

In several studies, human shoulder muscle activity data have been collected, both dynamically (Hawkes et al., 2019; Fice et al., 2021) and isometrically (Nadon et al., 2016), in a variety of postures. Because dynamic muscle load sharing has been shown to differ from isometric load sharing in other body regions (Ólafsdóttir et al., 2015), it is important to build the model using dynamic data to predict muscle activation for drivers in evasive maneuvers. In addition, a representative posture is important, as muscle lines of action and recruitment change in different postures (Nadon et al., 2016; Cudlip et al., 2018; Meszaros et al., 2018). Dynamic data of volunteers in a seated driver-like posture were recently collected for the purpose of being used in active FE-HBMs (Fice et al., 2021). In this study, approved by the Swedish Ethical Review Authority, eight male and nine female volunteers, after providing written informed consent, had a dynamic external load applied to their elbow in eight different directions and were instructed to return to the initial position as fast as possible. Muscle activity was recorded from 13 shoulder muscles using surface electromyography (EMG) electrodes. EMG measurements were normalized using maximum voluntary isometric contractions (MVIC). The authors of the volunteer study (Fice et al., 2021) provided the data used for simulation in this study.

The main objective of this study was to take an important step toward creating an active FE-HBM that can predict driver kinematics during evasive maneuvers by developing a method to control shoulder muscles, including intermuscular load sharing, based on measured muscle activity data from volunteers. The aim was to create a shoulder muscle controller capable of predicting human-like elbow kinematics when exposed to dynamic elbow loading.

## Methods

To implement and evaluate the new shoulder muscle controller concept, active shoulder muscles were introduced to the SAFER HBM. A new controller was implemented and evaluated by comparing detailed elbow kinematics of the model and the volunteers. A sensitivity study was conducted to study the effect of varying the gains of the controller.

### Submodel

**FIGURE 1:**
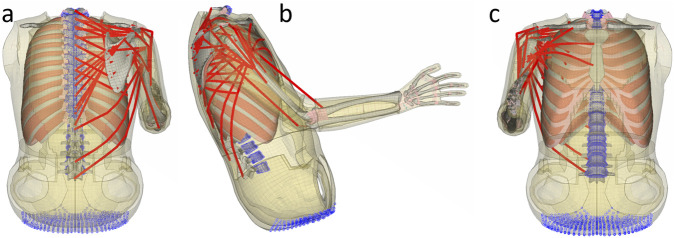
Submodel with updated shoulder muscles: **(a)** posterior, **(b)** lateral, and **(c)** anterior view. Skin and adipose tissue are shown in yellow, passive muscle tissue in salmon, bone in gray, joints and cartilage in pink, and active muscle elements in red. Constraints are shown with blue circles.

A submodel consisting of the thorax and the right upper extremity, excluding the internal organs, was extracted from the SAFER HBM v10 (Figure 1). Other body parts included were the C7–L5 vertebrae and buttocks/lower abdomen soft tissue; however, the pelvis was excluded. The acromioclavicular, sternoclavicular, and glenohumeral joints were modeled using six-degrees-of-freedom beams that allow rotation of the joint within a specified range while preventing translation. Surface-to-surface contact was modeled between the scapula and torso. In the simulations, the vertebrae were constrained in all translations and rotations, and the bottom surface of the buttock skin was constrained in the vertical and lateral translational directions, as shown in Figure 1. Simulations were run in LS-DYNA version MPP d R9.3.0 (LSTC, Livermore, United States), and pre- and postprocessing were done using ANSA/META (BETA CAE Systems, Switzerland), v 21.0.1, LS-PREPOST v 4.6.20 (LSTC, Livermore, United States), and MATLAB v R2020b (MathWorks, Massachusetts, United States).

### Input and evaluation data

**FIGURE 2:**
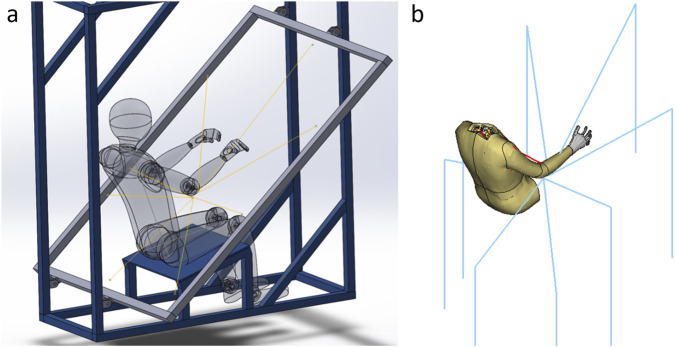
**(a)** Computer-aided design (CAD) model of the experimental setup (Fice et al., 2021). Note that the back support and chest strap restraints used in the experiment are omitted here; **(b)** FE-HBM submodel and the cable/pulley system (blue) for all eight loading directions (flexion, extension, adduction, abduction, flexion adduction, flexion abduction, extension adduction, and extension abduction). Only one of these eight was included per simulation.

EMG and kinematic data from a volunteer test series (Fice et al., 2021) were used as input to the controller and in the evaluation of the model. In the volunteer tests, eight male and nine female volunteers were seated in a test rig, presented schematically in Figure 2, with their torso constrained and subjected to elbow loading in eight directions (flexion, extension, adduction, abduction, flexion adduction, flexion abduction, extension adduction, and extension abduction). The subjects were secured to the seat back using a strap around the ribcage. Loading on the elbow was introduced by dropping an 8 kg weight attached to the elbow through a fixture and a cable/pulley system. The volunteers were instructed to return to the original position as soon as possible after the weight had been dropped. They were unaware of the direction of loading in each trial, and the directions were randomized. Muscle activity was measured in 13 muscles using surface electrodes. Kinematics were captured using video tracking. Initially, MVIC tests were performed, and subsequent EMG measurements were normalized by the obtained values. In the current study, spatial tuning patterns (STPs) were built by extracting peak EMG values for all eight loading directions and for all 13 muscles in all male and female subjects, from the first 1,000 ms of loading. The baseline muscle activity level was extracted prior to applying the loading. Furthermore, for biceps and triceps, normalization data were collected with shoulder MVICs. However, because these two muscles are elbow prime movers rather than shoulder prime movers, the normalization data were erroneous (these two muscles were not tensed to their maximums in the original MVICs), and data from these two muscles were excluded from the original publication (Fice et al., 2021). To allow for inclusion of original data for these two muscles in the muscle controller, a complementary study, using an identical MVIC protocol as in the original study, where 14 (9 male and 5 female) subjects were tested with both shoulder (in maximum abducted, adduction, extension, and flexion for the upper arm in different postures) and elbow (in maximum extension and flexion) MVICs (in accordance with the ethical application 2022-03970-01 that was approved by the Swedish Ethical Review Authority). From these new tests, ratios between biceps brachii and triceps brachii activity in shoulder and elbow MVICs were calculated (1.56 for biceps and 1.82 for triceps), and the original volunteer biceps brachii and triceps brachii data (normalized using shoulder MVICs) from all female and male subjects were scaled by dividing by these ratios.

**FIGURE 3:**
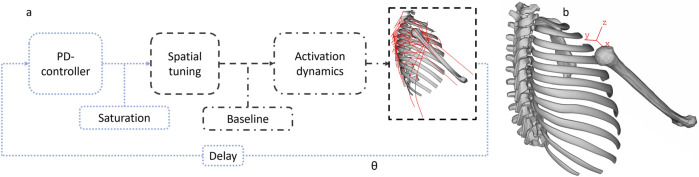
Illustration of APF controller logic **(a)**. The blue dotted lines represent signals on a model level; the dashed gray lines are muscle group-specific signals. The reference angle, θ, is measured in the local coordinate system in **(b)**. Local coordinate system for angle deviation and spatial tuning **(b)**.

The kinematics of the upper arm were represented by the translation of a point approximately at the elbow joint center and were transformed to a plane perpendicular to the initial humerus orientation (Fice et al., 2021) (the YZ plane depicted in Figure 3 and shown in relation to the model), using a polar coordinate representation. The average and standard deviation (SD) of volunteer elbow kinematics were calculated, for each time step, using polar representation, that is, average displacement magnitude and average displacement direction. SD ellipses were created, as described by Shaw et al. (2006). Kinematics were evaluated for 800 ms, as this was estimated as the approximate time it took most of the volunteers to first return to the initial posture, although significant oscillation continued afterward. SD ellipses were included in the kinematic plots twice: time at peak displacement and at 800 ms. The average and SD of peak displacement and time-to-peak (TTP) displacement were calculated from the individual peak displacements and TTPs.

Using the two methods of calculation presented above elicits two different peak displacement values: one is the peak of the average displacement, and the other is the average peak displacement of the subjects, where the peak of the average displacement will be lower than the average of the peaks. For the first method, TTP will be one number, without any SD, while for the second method, the TTP is calculated for each subject, and thus both average and SD can be calculated.

### Muscle implementation

The shoulder muscle package of the SAFER HBM v10 was used as a foundation for the muscle implementation (Östh et al., 2015). In addition, muscles not already included as active muscles, which are involved in upper extremity movement according to Betts et al. (2013), were either added to the model or converted from passive elements to active elements. Muscle data are presented in Supplementary Table S1. The updated shoulder muscle package consisted of 179 1D elements; see Figure 1 for a graphical presentation. These elements were all modeled using an LS-DYNA Hill-type MAT_156 muscle model (LS-DYNA® KEYWORD USER'S MANUAL, 2016).

The bi-articulate muscles, that is, triceps and biceps brachii, have been split into one portion that belongs to the shoulder controller and one portion that belongs to the elbow controller. Splitting the muscles was done by equally splitting the physical cross-sectional area (PCSA) of the short head of the triceps and long head of the biceps between the shoulder and elbow controller. The biceps short head also spans both joints. As the lever arm over the shoulder joint is small, the short head PCSA was assigned only to the elbow controller. However, it should be noted that this is an approximation, and how this split should be performed is of importance, as the function of the muscle also depends on co-activation with surrounding muscles.

Muscles that wrap around structures, such as the serratus anterior, were modeled with multiple 1D elements along the length. The same force was ensured in all connected elements by using a functionality that averages the force in the element chain on all elements (PART_AVERAGE). In addition, the nodes not representing the muscle insertions, that is, all nodes in the element chains except for the two end nodes, were constrained in the flesh solid elements using CONSTRAINED_INTERPOLATION. The load was distributed to the solids on translational degrees of freedom and transferred to the nodes in closest proximity to the muscle nodes.

To check for the correct routing of the muscles, each muscle was activated in isolation, and the displacement was compared with its anatomical function (Betts et al., 2013; Marieb and Hoehn, 2019). In simulations evaluating muscles with a theoretical effect on the humerus, the scapula was prevented from moving to isolate the effect on the humerus.

### Implementation of muscle controllers

Fundamentally, a shoulder has two aspects that must be controlled: scapula translations and glenohumeral rotations. To control the glenohumeral rotations, the glenohumeral joint muscles were controlled using angular position feedback (APF), using the approach previously described by Ólafsdóttir et al. (2019). To control the scapula, all muscles spanning from the torso or the spine to the scapula were controlled using muscle length feedback (MLF), a method of emulating the muscle stretch reflex (Ólafsdóttir et al., 2019).

The APF controller uses a proportional–derivative (PD) controller that responds to an angular deviation of a reference vector, spanning from the proximal to the distal end of the humerus, between the lateral and medial epicondyles, in a local coordinate system, with the X-axis in the humerus longitudinal direction and the Y-axis in the lateral direction, shown in Figure 3. In the present study, the coordinate system of the submodel is constrained to the ground. While the PD controller is allowed to work in a larger range, the response is saturated to 1 (using min/max functions), as the Hill-type MAT_156 muscle model works with normalized signals (LS-DYNA® KEYWORD USER'S MANUAL, 2016). The intermuscular load sharing is given by experimentally derived STPs (Fice et al., 2021). Spatial tuning is performed in a plane perpendicular to the initial humerus orientation in the same coordinate system as the PD controller, that is, in the YZ plane. For each direction, the experimentally derived STPs were rescaled/normalized with the highest reported activity among the muscle-specific MVIC normalized EMG signals, limited to muscles included in the controller. The differences between the STPs from experiments and those rescaled for the controller are shown in Supplementary Figure S1. Subsequently, the baseline activity (Fice et al., 2021) was added to the signal (Supplementary Table S3). The baseline activity was taken from the normalized EMG data before any loading. In the feedback loop, a delay (10 ms for the MLF controllers and 30 ms for the APF controller) was introduced to the angle error signal going to the PD controller, to represent the time delay between sensory input and the signal reaching the surface of the muscle (Ólafsdóttir et al., 2019) (Supplementary Table S2). Activation dynamics were added to the signal to replicate the delay between a muscle receiving an action potential and contracting (Winters and Stark, 1985; Ólafsdóttir et al., 2019). The details are presented in Supplementary Table S2.

The controller gains were based on the response of the passive model. The hypothetical control error signal from the passive model was extracted, and the proportional (P) and derivative (D) gains of the PD controller were set such that these would each produce a maximum control signal of 1 because muscle activation is not intended to exceed unity in the Hill-type muscle model (Table 1, baseline).

**TABLE 1:** Controller gains.

Gain	APF-baseline	APF-high gain	APF-low gain	MLF (Ólafsdóttir et al., 2019)
P	1.6 [1/rad]	2.4 [1/rad]	0.8 [1/rad]	0.5 [1/mm]
D	150 [1/rad ms^−1^]	225 [1/rad ms^−1^]	75 [1/rad ms^−1^]	5 [1/mm ms^−1^]

In the MLF control system, each muscle is controlled with a separate PD controller, responding to lengthening of the muscle part (Ólafsdóttir et al., 2019). Some muscles were modeled using several parts across the width, and some muscle parts were modeled using several 1D muscle elements in series. For parts with several elements in series, the entire element chain’s lengthening was used as input to the PD. Just as for the APF controller, transmission delay and activation dynamics were added to the controller loop (Supplementary Table S2). MLF gains were based on values from Ólafsdóttir et al. (2019) and are presented in Table 1, while baseline activity from Östh et al. (2014), presented in Supplementary Table S3, was used.

### Verification of the kinematics

The kinematic performance of the model was verified by simulating the volunteer experiments from which the STPs were derived. The elbow joint was extended to a 130° angle in a pre-simulation and then constrained to that angle to match the lower arm position from Fice et al., 2021. In the experiments, a load on the elbow was introduced by dropping an 8 kg weight attached to the elbow with a cable/pulley system. With this setup, the measured load depends on the subject’s reactions. To include this effect, the setup was modeled with a weight drop through a pulley system, with routing points in the same locations as in the experiment, as seen in the schematic presentation in Figure 2. The weight was modeled as an 8 kg point mass. An initial velocity of 0.77 m/s was added to the mass to mimic slack in the release mechanism from the volunteer tests.

Peak elbow displacement and TTP, as well as kinematic traces with and without active muscle control, were compared with volunteer results. For the volunteers, the peak displacement and TTP were calculated from the subjects’ individual peaks and TTPs, while the kinematic traces were calculated by averaging the subjects’ displacements for each time step.

### Sensitivity study

The APF PD controller gains were varied to study the effect of gains. The P and D gains were then increased and decreased by 50% from the baseline value. The two higher and lower gains were run in pairs to show any combined effect. In total, seven sets of gains plus one passive model were simulated in eight directions, resulting in 64 simulations.

## Results

When each muscle was activated in isolation, most muscles performed the same movements as described in the literature (Supplementary Table S1). Some muscles, such as the latissimus dorsi, showed slightly different functions.

### Detailed kinematics

**FIGURE 4:**
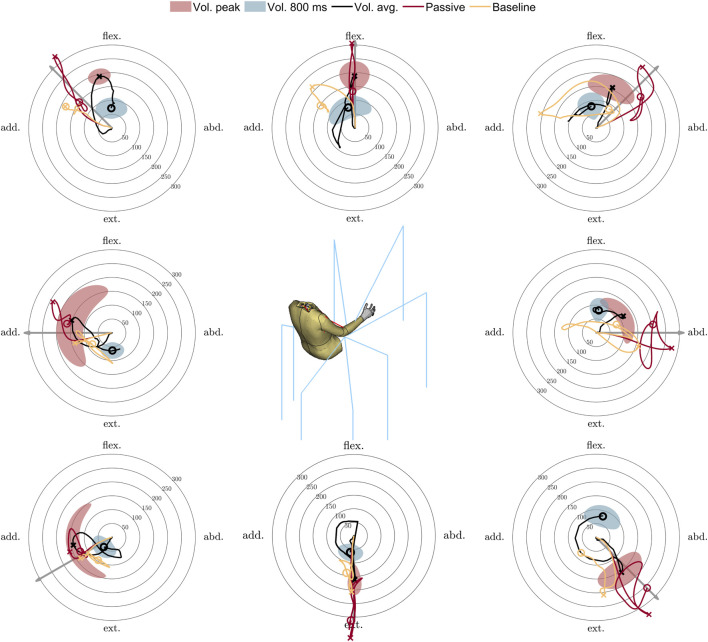
HBM with baseline gains (yellow), passive HBM (red), and average volunteer (black) elbow kinematics (Fice et al., 2021), relative T1 for volunteers and ground for HBM, measured in a plane perpendicular to initial humerus orientation (plane normal to first axis in Figure 3). Filled areas highlight the volunteer average ± 1 SD, in polar coordinates. The cross markers on the lines indicate the peak, and the circles indicate 800 ms. The gray arrow shows the loading direction. Units are mm.

The volunteers did not follow a straight path when returning to the original position, for any of the directions, nor did the active model, as shown in Figure 4. Neither the volunteers nor the simulation models could return to the exact initial position. At 800 ms, the volunteers were within 100 mm of the initial position for all loading directions, while the active model (Baseline) was up to 200 mm from the initial position in the flexion adduction load case (Figure 4). In all directions, the active simulation model moves in the same direction as the volunteers immediately after peak elbow displacement. Late in the process, the model deviated from the volunteer results. The active model had a similar magnitude in peak elbow displacement as the volunteers, while the passive model had larger peak elbow displacements for all directions. In three of the directions, adduction, extension, and extension adduction (Figure 4), the peak elbow displacement of the active model fell within the volunteer SD ellipse, while the passive model was only inside the peak ellipse for extension adduction (Figure 4), where the elbow moves toward the torso. The active model displacement at 800 ms did not fall within the SD ellipse in any of the simulations.

### Sensitivity study

**FIGURE 5:**
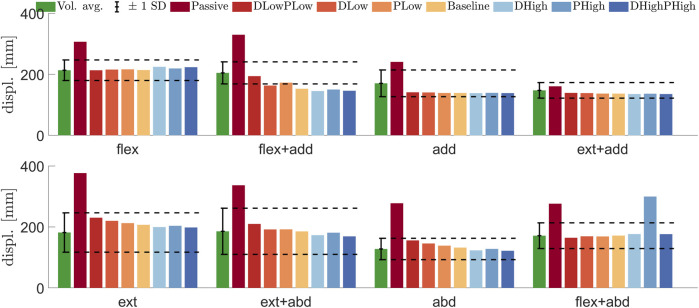
Average ± 1 SD peak displacement for volunteers (Fice et al., 2021) (green) compared with time to peak for simulation models: passive (Passive), both gains low (DLowPLow), low D and baseline P (DLow), baseline D and low P (PLow), baseline gains (Baseline), high D and baseline P (DHigh), baseline D and high P (PHigh), and both gains high (DHighPHigh) in the eight loading directions: flexion (flex), flexion adduction (flex + add), adduction (add), extension adduction (ext + add), extension (ext), extension abduction (ext + abd), abduction (abd), and flexion abduction (abd). The color scale for the model plots was created with ColorBrewer (Cobeldick, 2021).

Active muscle control reduced the peak elbow displacement compared with the same model without muscle activation for all eight loading directions, as shown in Figure 5, except for the model with high P gains in flexion abduction. The peak displacement for the passive model was more than 1 SD from the volunteer peak displacement for all loading directions except extension adduction. The gain variation shows that for most simulations, increasing the P or D gains reduces the peak displacement. In extension adduction and pure adduction, the effect of varying the gains was minimal. For all loading directions except for flexion adduction and flexion abduction, all active models were within 1 SD from the volunteer peak elbow displacement. In flexion adduction, the two simulations with low P gain were within 1 SD from the volunteers, while the other gain combinations produced lower peak displacements.

**FIGURE 6:**
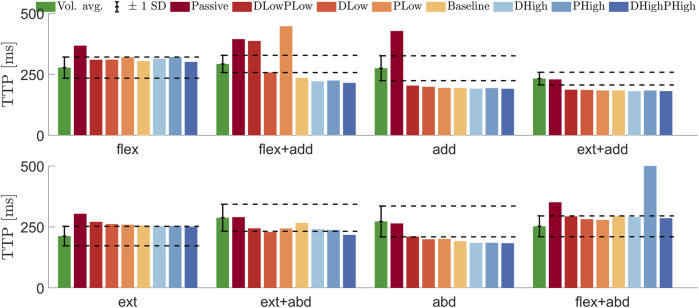
Average ± 1 SD time-to-peak displacement for volunteers (Fice et al., 2021) (green) compared with the time to peak (TTP) for simulation models: passive (Passive), both gains low (DLowPLow), low D and baseline P (DLow), baseline D and low P (PLow), baseline gains (Baseline), high D and baseline P (DHigh), baseline D and high P (PHigh), and both gains high (DHighPHigh), in the eight loading directions: flexion (flex), flexion adduction (flex + add), adduction (add), extension adduction (ext + add), extension (ext), extension abduction (ext + abd), abduction (abd), and flexion abduction (abd). The color scale for the model plots was created with ColorBrewer (Cobeldick, 2021).

The TTP for most directions was decreased with higher gains, as shown in Figure 6. For the passive model, the TTP was above the SD range for five of the cases, while for the other three (extension adduction, extension abduction, and abduction), the passive model TTP was within the SD range. The active models provided varying results with regard to TTP. In adduction and extension adduction, the TTP was below that of the volunteers for all active models, whereas in extension, all active models had a TTP higher than that of the volunteers. In flexion adduction, the models produced TTPs both above (low P gains) and below (baseline and high gains) the SD range.

In summary, the sensitivity was introduced to present model sensitivity and guide future work. However, if one is to choose PD controller gains from this sensitivity study, the version with low D gains and baseline P gains (DLow) provided the most reasonable responses when peak displacements and TTP were evaluated.

## Discussion

The aim of this study was to create a shoulder muscle controller capable of predicting human-like elbow kinematics when exposed to dynamic elbow loading. The shoulder muscle feedback controller was designed to include intermuscular load sharing and be based on recorded human muscle activation data from volunteer experiments. The current study focused on the implementation of a shoulder muscle controller into SAFER HBM and was evaluated using a simplified setup. This shoulder muscle controller is intended for use in an FE-HBM to simulate driver kinematics during evasive maneuvers. While all development work was done using SAFER HBM v10, the method of controlling the muscles is model agnostic.

### Muscle routing

Not all muscles performed the same movements as described in the literature (Supplementary Table S1) when each muscle was activated in isolation. Some muscles, such as the latissimus dorsi, showed slightly different functions. It is described in the textbooks as an arm adductor, an arm extensor, a spine stabilizer, and a humerus internal rotator (Marieb and Hoehn, 2019), but it did not rotate the humerus internally in the simulation. To address this, not only should the muscle origins and insertions be carefully reviewed, but bone geometry for the humerus, scapula, clavicle, and shoulder joints should also be updated.

### Controller gains and performance

Controller performance was verified by comparing the model kinematics to volunteer kinematics. Peak elbow displacements and TTP as predicted by the active model were compared with those predicted by the passive model, and with those recorded in the volunteer experiments. If the active muscle control implementation is effective, a reduced peak elbow displacement and TTP can be expected. The muscle controller reduces peak displacement for all directions, meaning that for each direction, the muscles are routed and activated in a directionally sound manner. Peak elbow displacements were predicted within 1 SD for all eight directions. Time-to-peak displacement was predicted within 1 SD for four directions. This successfully demonstrates that recorded human muscle activation data can be used to determine intermuscular load sharing for an active shoulder muscle controller.

In loading directions incorporating adduction, the upper arm contacts the torso, which restricts further movement. This could explain why changing the gains had very little effect on peak displacement in adduction and extension adduction, as shown in Figure 5. For these cases, the internal relationship between the humerus and torso, together with the torso boundary condition, becomes more important than for the cases where the arm does not contact the torso. The setup was simplified to reduce simulation time, such that the spine and buttocks were constrained from movement, omitting the test rig from the simulation. The internal positions of the arm and torso have been based on the experimental setup. If the load case should be used for validation, a more detailed simulation environment and HBM positioning might be required.

The implemented controller is intended to model reflex behavior, an early response to stimuli (Scott, 2012), which is a response considered representative of relaxed vehicle occupants who are suddenly exposed to autonomous braking or steering of the vehicle. Later in the process, muscle activation can be attributed to voluntary control rather than the reflexes (Latash, 2008). This could explain why the model matches the volunteers better before and immediately after peak displacement, as shown in Figure 4, which occurs approximately 200–300 ms after loading onset, as shown in Figure 6, compared with later in the process. Data show that voluntary control flexibly alters muscle control gains to reduce task-relevant errors rather than optimize for a path to reach the goal (Scott, 2012). In the experimental data, the volunteers were instructed to return to the center quickly, although instructions on how to return were not provided. This could explain the large directional SDs seen in Figure 4.

In conclusion, an active shoulder muscle controller was implemented in an FE HBM. The intermuscular load sharing was based on recorded muscle activity data from volunteers, and the model was evaluated by comparing elbow kinematics to data from volunteers exposed to dynamic elbow loading in eight directions. The model successfully predicted peak elbow displacement for all directions. Thus, the controller is ready to be integrated and evaluated in a full-body FE-HBM driver exposed to evasive maneuvers.

## Limitations

The gains of the controller were tuned and evaluated only for a limited set of elbow-loading directions and must be optimized in future implementations. As the simulated case is different from a driving scenario where the hands would be supported by the steering wheel, a different load case should be used for that optimization. The gains used in this study would most likely perform differently in another load case, as human reflexes are context dependent (Forbes et al., 2013). A sensitivity study was performed to investigate the effect of controller gains. The low controller gains showed better agreement with volunteer results for peak elbow displacement, although simultaneously reducing the model’s capacity to return to the original position. It remains to be determined what the model should be compared with in the optimization process. With this setup, the geometry of the model and intermuscular load sharing can be evaluated.

One limitation in the current study was the use of a thorax–upper-extremity submodel with constraints on the spine and buttocks, and a simplified experimental setup. Although the volunteer upper torso motions were minimal in the original experiments, the use of this submodel excludes trunk dynamics and whole-body interactions, which may influence muscle coordination and stability under realistic driver maneuver conditions. In braking or steering maneuvers, head and torso inertia would introduce forces to the shoulder, instead of forces introduced in the elbow as in this study. Because these two scenarios are similar mechanically, the simplified approach was used instead, as this allowed for an experiment where the force magnitude and direction could easily be controlled. However, prior to the use of the shoulder model in a full-body setup, such as an updated SAFER HBM or another HBM, the implementation must be tuned and validated with the full-body HBM subjected to vehicle accelerations.

Forces from vehicle decelerations, due to body inertia, were not included in the original experiments or in the simulations carried out in this study. These inertia forces will add load to the shoulder in a similar way as the cables loaded the shoulder via the elbow attachments, which was the focus of this study. However, vehicle decelerations will likely provide for shoulder loading with different durations and amplitudes. While the amplitude used in the original experiments was chosen so that volunteer elbow rapidly moved in the direction of the loading, while the volunteer reaction forces were sufficient to make the motion come to a full stop, the elbow joint forces in a driver of a vehicle when subjected to braking or steering maneuvers are likely somewhat lower (forces estimated from steering wheel forces are presented in Östh et al., 2013). These differences call for additional evaluations of the shoulder joint model when it is integrated into a full-body HBM.

The implemented controller does not explicitly account for voluntary muscle contractions (Fice et al., 2020), where the driver braces themself on the steering wheel in anticipation of an impact or to avoid excessive motions in evasive steering or braking (Hault-Dubrulle et al., 2011b). In a previous version of the active full-body SAFER HBM, bracing was successfully included by moving the target posture for the elbow joint, while the shoulder joint aimed at maintaining the initial posture (Östh et al., 2014). The same approach may be adopted and evaluated with the updated shoulder controller in the full-body FE-HBM implementation. The developed controller is intended to model reflex muscle activation, and development work would be needed to model voluntary actions such as, for instance, turning the steering wheel. Additionally, the STPs from volunteer experiments were built using peaks from the first 500 ms, which could explain why the model was less effective at capturing kinematics later in the simulation. Using time windows for the STPs could be one method to overcome this, but because the controller is intended to model the reflex behavior, the STPs from the first 500 ms were used.

To allow for muscles to wrap around structures such as the ribcage, several 1D elements have been connected in series. Allowing the active muscles to wrap around such structures is important to model the correct line of action on both origin and insertion of the muscles. Other research groups have solved similar problems, for instance, in the elbow, by routing the muscles through via-points that rigidly connect to bony structures (Kleinbach et al., 2017). The via-point approach can ensure that the lines of action at the nominal position are correct, but once the model starts moving, the lines of action may change substantially. In the elbow joint, which allows for rotation around one axis only, a carefully selected placement of the via-point can avoid this issue. However, given the considerable flexibility of the shoulder joint, the lines of action could become severely distorted if a via-point method is used. In future work on the shoulder, other approaches may be adopted, for example, the via-ellipse approach for muscle routing presented by Hammer et al. (2019). Constraining the muscles in the flesh could also lead to a distortion of lines of action; however, because the flesh is more flexible than bone, this is less of a problem. The wrapping was flexible enough to reasonably handle the simulations in this study. If the model is to be used in situations in which the arm moves enough to distort the lines of action, even with the proposed wrapping method, applying an approach with combined beam and solid elements may be more appropriate (Hedenstierna et al., 2008) or the inclusion of muscle wrapping as suggested by Iwamoto and Nakahira (2012) or included in more recent muscle models.

The coordinate system for the reference vector and spatial tuning is constrained to the ground in the submodel. In a full model, this could lead to artificial activation of muscles, for instance, if the full model undergoes rigid body rotations. The controller will perceive such a rotation as a deviation from reference, even though the internal positions of body regions have not changed. To overcome this problem, the reference coordinate system will be constrained to the torso in full-body FE-HBM implementations (Larsson et al., 2019).

## Data Availability

The original contributions presented in the study are included in the article/Supplementary Material, further inquiries can be directed to the corresponding author.
