# Supplementary material for: Development of a shoulder muscle feedback controller for human body models
Source: Front Bioeng Biotechnol. 2026 Mar 5;14:1694396. doi: 10.3389/fbioe.2026.1694396 (PMC12999849; doi:10.3389/fbioe.2026.1694396)
Supplement: Supplementary file 1 [file Supplementaryfile1.docx]

Supplementary

Table S1. Muscles included in the model, muscle grouping, origin, insertion, number of elements and anatomical function.

| Muscle | In SAFER v10 | Origin^(a)^ | Origin model | Insertion^(a)^ | Insertion model | No. parts/elements | PCSA [cm^2^] | Grouping | Function – theory^(b)^ | Function - model |
| --- | --- | --- | --- | --- | --- | --- | --- | --- | --- | --- |
| Pectoralis major^(c)^ (PecMaj) | Active | Clavicle; sternum; cartilage of certain ribs (1-6 or 1-7); aponeurosis of external oblique muscle | Clavicle; sternum (x2) | Greater tubercle of humerus | Ant. Humerus, 0.22-0.29 of HL^(h)^ | 3/31 | 15.9 | PecMaj | Humerus flexion, adduction, internal rotation | Humerus flexion, adduction, internal rotation |
| Latissimus dorsi^(c)^ (LatDors) | Active | Thoracic vertebrae (T7—T12); lumbar vertebrae; lower ribs (9-12); iliac crest | T12, L3, L5 | Intertubercular sulcus of humerus | Post. humerus, 0.23-0.29 of HL^(h)^ | 3/30 | 13.9 | LatDors | Humerus extension, adduction, internal rotation | Humerus extension, adduction |
| Deltoid anterior^(c,f)^ (AntDelt) | Active | Clavicle; acromion; spine of scapula | Clavicle, acromial end | Deltoid, tuberosity of humerus | Ant. Humerus, 0.35 of HL^(h)^ | 1/2 | 7.9 | AntDelt | Humerus abduction, flexion, internal rotation | Humerus abduction, flexion |
| Deltoid middle^(c,f)^ (MidDelt) | Active | Clavicle; acromion; spine of scapula | Acromion | Deltoid, tuberosity of humerus | Med. Humerus, 0.35 of HL^(h)^ | 1/2 | 16.3 | MidDelt | Humerus abduction | Humerus abduction |
| Deltoid posterior^(c,f)^ (PosDelt) | Active | Clavicle; acromion; spine of scapula | Acromion; spine of scapula | Deltoid, tuberosity of humerus | Med. – post. Humerus, 0.35-0.52 of HL^(h)^ | 3/6 | 9.3 | PosDelt | Humerus abduction, extension, external rotation | Humerus abduction, extension |
| Subscapularis^(c)^ | Passive 3D layer (not connected to humerus) | Subscapular, fossa of scapula | Subscapular, fossa of scapula/medial border | Lesser, tubercle of humerus | Lesser, tubercle of humerus | 4/4 | 14.1 | TerMaj | Humerus internal rotation | Humerus internal rotation, adduction |
| Supraspinats ^(c),^ (SupSpin) | Passive | Supraspinous fossa of scapula | Supraspinous fossa of scapula/superior angle | Greater tubercle of humerus | Greater tubercle of humerus | 1/2 | 4.8 | SupSpin | Humerus abduction | Humerus abduction |
| Infraspinatus ^(c)^  (InfSpin) | Passive | Infraspinous fossa of scapula | Infraspinous fossa of scapula/medial border | Greater tubercle of humerus | Greater tubercle of humerus | 2/2 | 11.9 | InfSpin | Humerus external rotation | Humerus external rotation, extension |
| Teres major^(c)^  (TerMj) | Active | Posterior surface of scapula | Inferior angle of scapula | Intertubercular sulcus of humerus | Ant. Humerus, 0.19 of HL^(h)^ | 1/1 | 2.5 | TerMaj | Humerus adduction, extension, internal rotation | Humerus adduction, extension |
| Teres minor^(c)^ | Passive | Lateral border of dorsal scapular surface | Medial border of scapula (between spine and inferior angle) | Greater tubercle of humerus | Greater tubercle of humerus | 1/1 | 3.7 | InfSpin | Humerus adduction (weak), external rotation | Humerus adduction, external rotation |
| Coracobrachi-alis^(c)^ | Active | Coracoid process of scapula | Coracoid process of scapula | Medial surface of humerus shaft | Ant. humerus, 0.45 of HL^(h)^ | 1/1 | 2.4 | Bic | Humerus flexion, adduction | Humerus adduction |
| Triceps long head^(g)^  (Tri) | Active (controlled by elbow controller) | Infraglenoid tubercle of scapula; posterior shaft of humerus; posterior humeral shaft distal to radial groove | Lateral border (superior part) | Olecranon process of ulna | Olecranon process of ulna | 1/1 | 2.2^(g)^ | Tri | Elbow extension, humerus extension | Humerus extension |
| Subclavius^(e)^ | Not included | First rib | Anterior surface of first rib | Inferior surface of clavicle | Inferior surface of clavicle | 1/1 | 4.4 | MLF | Clavicle depression | Clavicle depression |
| Pectoralis minor^(d)^ | Not included | Anterior surface of rib 3-5 | Anterior surface of ribs 3-5 | Coracoid process of scapula | Coracoid process of scapula | 3/3 | 3.5 | MLF | Scapula protraction | Scapula protraction |
| Serratus anterior^(d)^ | Passive | Muscle slips from ribs 1–8 (or 9) | Anterior/lateral surfaces of ribs 1-8 | Anterior surface of vertebral border of scapula | Medial border of scapula | 8/74 | 15.7 | MLF | Scapula protraction | Scapula protraction |
| Trapezius^(d)^ | Passive (upper trapezius active and included in neck controller) | Occipital bone; ligamentum nuchae; spinous process of C7, all thoracic vertebrae | T2, T3, T4 (mid), T6-T12 (low) | Acromion and spine of scapula; clavicle | Acromion and spine of scapula | 3/3 (mid), 7/7 (low) | 16.1 | MLF | Scapula retraction (mid), depression (low) | Scapula retraction, scapula depression (retraction) |
| Rhomboid major^(d)^ | Passive | Spinous process of: T2–T5 | T2-T6 | Medial border of scapula | Medial border of scapula | 5/5 | 3.8 | MLF | Scapula retraction | Scapula retraction (elevation) |
| Rhomboid minor^(d)^ | Passive | Spinous process of: C7 and T1 | C7, T1 | Medial border of scapula | Medial border of scapula | 2/2 | 1.0 | MLF | Scapula retraction | Scapula retraction (elevation) |
| Biceps brachii short head (shoulder)^(g)^  (Bic) | Active (controlled by elbow controller) | Coracoid process; tubercle above glenoid cavity | Coracoid process | Radial tuberosity | Radial tuberosity | 1/2 | 1.1 | Bic | Elbow flexion, humerus flexion | Humerus flexion, adduction |

(a) Betts et al. (2013)

(b) Marieb and Hoehn (2019)

(c) Holzbaur et al. (2007),

(d) Altobelli et al. (2008)

(e) Garner et al. (2001) (Subclavius only)

(f) Aluisio et al. 2003 (Deltoid PCSA distribution)

(g) Bi-articluate muscles, prime movers of elbow joint, biceps and triceps, assumption 50% of long/short head belong to shoulder controller (Murray et al. 2000).

(h) For muscles attaching to the humerus, where anatomical features are not available in the model, placement along the humerus length (HL) (measured from the most proximal node on the humerus) as portion of total HL together with approximate circumference placement was used to describe insertion point

Table S2. Activation dynamics parameters (Ólafsdóttir et al. 2019)

| Control strategy | Parameter | Value |
| --- | --- | --- |
| *APF and MLF* | *Time constant, neural excitation* - T_ne_* | 35 ms |
|  | *Time constant, muscle activation* - T_na,a_* | 10 ms |
|  | *Time constant, muscle deactivation* - T_na,d_* | 40 ms |
| * These constants were used in the two first order differential equations proposed by Winters and Stark (1985), presented in Equations (1) and (2), where u is the muscle controller signal, N_e_ an intermediate neural excitation level and N_a_ the excitation signal used in the muscle elements. | | |

| $\frac{dN_{e}}{dt}=\frac{\left( {u-N}_{e} \right)}{T_{ne}}$ | (1) |
| --- | --- |
| $\frac{dN_{a}}{dt}=\left\{ \begin{aligned} \frac{\left( N_{e}-N_{a} \right)}{T_{na,a}}, &N_{e}\geq N_{a} \\ \frac{\left( N_{e}-N_{a} \right)}{T_{na,d}}, &N_{e}<N_{a} \end{aligned} \right.$ | (2) |

Table S3. Baseline activity

| Muscle | Baseline activity (Fice et al. 2021) |
| --- | --- |
| SupSpin | 0.0253 |
| AntDelt | 0.0221 |
| MidDelt | 0.0089 |
| PosDelt | 0.0038 |
| PecMaj | 0.0138 |
| LatDors | 0.0184 |
| TerMaj | 0.0087 |
| Bic | 0.0063 |
| Tri | 0.0031 |
| InfSpin | 0.0141 |
| *MLF ^45^* | 0.0400 |

| 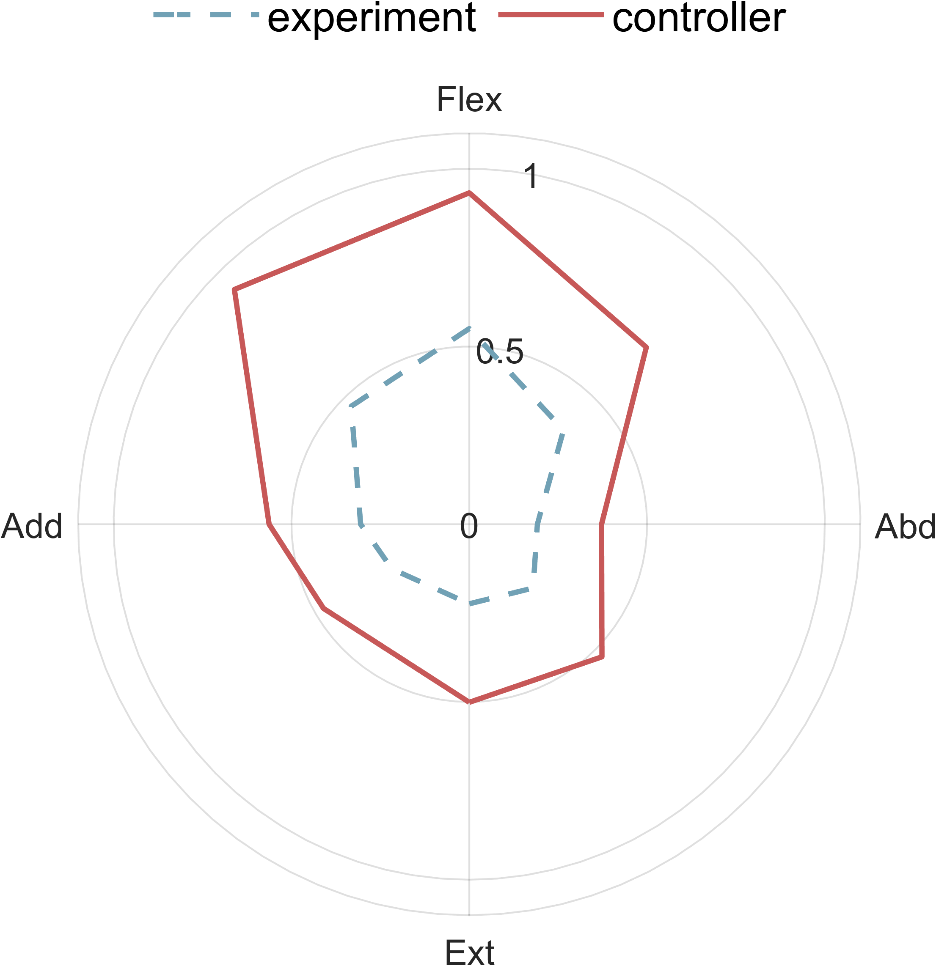 |
| --- |

| 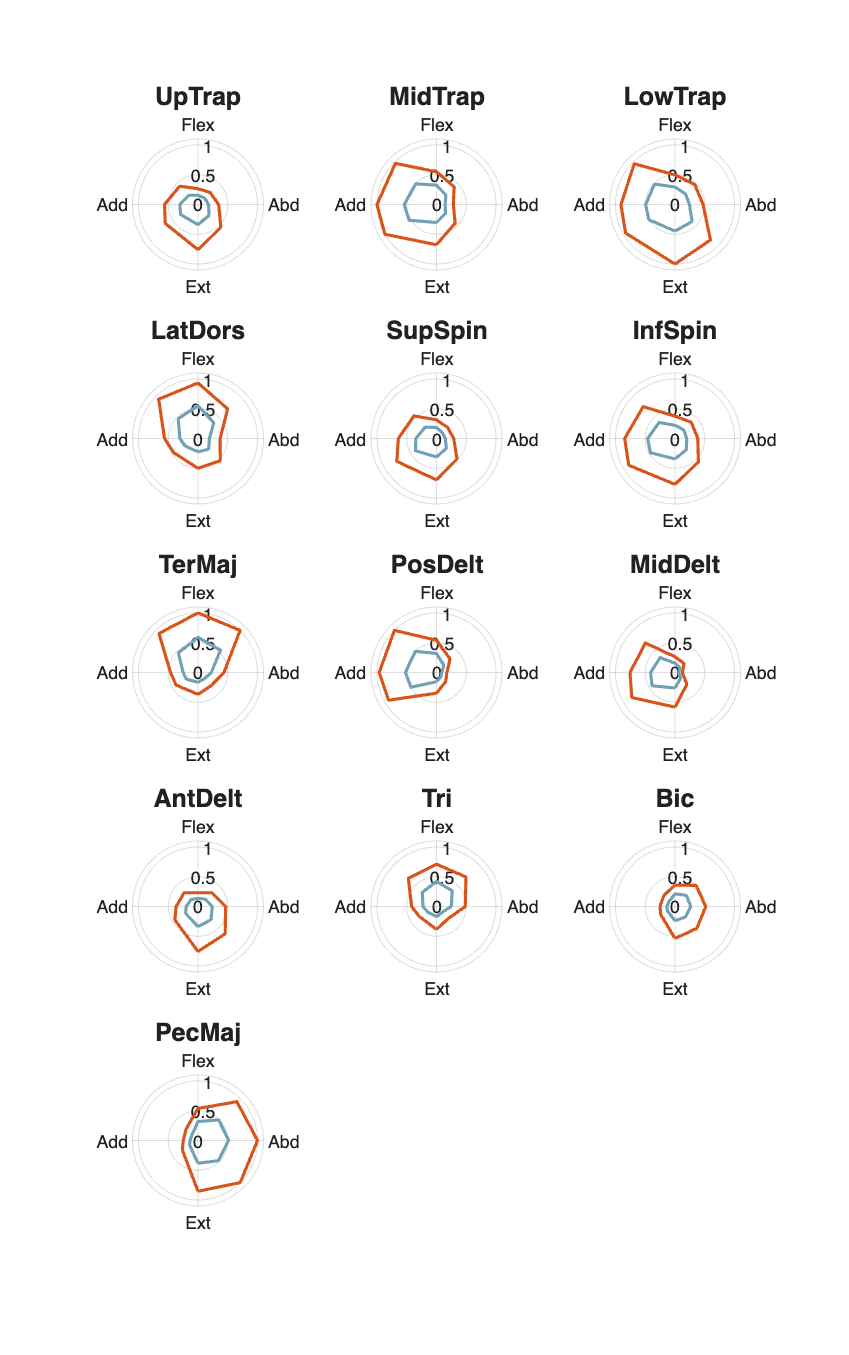 |
| --- |

| Fig. S1. STPs, blue curves are derived from experiments (Fice et al. 2021), red curves are rescaled for controller. |
| --- |

References

Altobelli G, Eng CM, Taylor AB, Gokhin D, Lieber RL, Ward SR. 2008. Scapulothoracic and glenohumeral muscle architecture in middle-aged individuals. Proceedings of the 54th Annual Meeting of the Orthopaedic Research Society.

Aluisio FV, Osbahr DC, Speer KP. 2003. Analysis of rotator cuff muscles in adult human cadaveric specimens. American journal of orthopedics (Belle Mead, NJ). 32 (3):124-129.

Betts JG, DeSaix P, Johnson E, Johnson JE, Korol O, Kruse DH, Poe B, Wise JA, Young KA. 2013. Anatomy and physiology.

Fice JB, Larsson E, Davidsson J. 2021. Dynamic Spatial Tuning Patterns of Shoulder Muscles with Volunteers in a Driving Posture. Frontiers in bioengineering and biotechnology. 9.

Garner BA, Pandy MG. 2001. Musculoskeletal model of the upper limb based on the visible human male dataset. Computer methods in biomechanics and biomedical engineering. 4 (2):93-126.

Holzbaur KR, Murray WM, Gold GE, Delp SL. 2007. Upper limb muscle volumes in adult subjects. Journal of biomechanics. 40 (4):742-749.

Marieb EN, Hoehn K. 2019. Human anatomy & physiology. 11th edition, Global edition. ed. Pearson Education Limited.

Murray WM, Buchanan TS, Delp SL. 2000. The isometric functional capacity of muscles that cross the elbow. Journal of biomechanics. 33 (8):943-952.
